# Genomic determinants of long-term cardiometabolic complications in childhood acute lymphoblastic leukemia survivors

**DOI:** 10.1186/s12885-017-3722-6

**Published:** 2017-11-10

**Authors:** Jade England, Simon Drouin, Patrick Beaulieu, Pascal St-Onge, Maja Krajinovic, Caroline Laverdière, Emile Levy, Valérie Marcil, Daniel Sinnett

**Affiliations:** 1 Research Centre, Sainte-Justine University Health Center, 3175 chemin de la Côte-Sainte-Catherine, Montreal, Quebec, H3T 1C5 Canada; 2 Departments of Pediatrics, Université de Montréal, Montreal, Quebec, H3T 1C5 Canada; 3 Departments of Nutrition, Université de Montréal, Montreal, Quebec, H3T 1C5 Canada

**Keywords:** Acute lymphoblastic leukemia, cancer survivors, genetic determinants, cardiometabolic complications, genetic association study, extreme phenotype, obesity, dyslipidemia, insulin resistance, hypertension

## Abstract

**Background:**

While cure rates for childhood acute lymphoblastic leukemia (cALL) now exceed 80%, over 60% of survivors will face treatment-related long-term sequelae, including cardiometabolic complications such as obesity, insulin resistance, dyslipidemia and hypertension. Although genetic susceptibility contributes to the development of these problems, there are very few studies that have so far addressed this issue in a cALL survivorship context.

**Methods:**

In this study, we aimed at evaluating the associations between common and rare genetic variants and long-term cardiometabolic complications in survivors of cALL. We examined the cardiometabolic profile and performed whole-exome sequencing in 209 cALL survivors from the PETALE cohort. Variants associated with cardiometabolic outcomes were identified using PLINK (common) or SKAT (common and rare) and a logistic regression was used to evaluate their impact in multivariate models.

**Results:**

Our results showed that rare and common variants in the *BAD* and *FCRL3* genes were associated (*p*<0.05) with an extreme cardiometabolic phenotype (3 or more cardiometabolic risk factors). Common variants in *OGFOD3* and *APOB* as well as rare and common *BAD* variants were significantly (*p*<0.05) associated with dyslipidemia. Common *BAD* and *SERPINA6* variants were associated (*p*<0.05) with obesity and insulin resistance, respectively.

**Conclusions:**

In summary, we identified genetic susceptibility loci as contributing factors to the development of late treatment-related cardiometabolic complications in cALL survivors. These biomarkers could be used as early detection strategies to identify susceptible individuals and implement appropriate measures and follow-up to prevent the development of risk factors in this high-risk population.

## Background

Childhood acute lymphoblastic leukemia (cALL) represents one third of all pediatric cancers [1]. Better understanding of the disease and treatment optimization over the last few decades has led to remarkable cure rates reaching 85% [2]. However, this therapeutic success comes at a substantial price since 60% of survivors currently face treatment-related long-term complications [3]. Children with cALL are exposed to chemo- and radiotherapy during a critical period of their development and thus have a greater risk of developing obesity [4], insulin resistance [2, 5], hypertension (HTN) [2, 6] and dyslipidemia [2], forming a metabolic syndrome (MetS) cluster [2]. These late treatment effects are worrisome since people affected by the MetS are at higher risk of atherosclerotic vascular disease [7], type 2 diabetes [8], and stroke [7]. The causes of these complications in cALL survivors remain unknown, but exposition to corticoids, methotrexate and cranial radiotherapy has been reported as contributing factor [9–12].

In the general population, accumulating evidence indicate that nutrition has an important influence on MetS susceptibility and treatment response [13–17]. Furthermore, several susceptibility loci and genes are linked to MetS occurrence [13]. For instance, 20-40% of the variance of arterial blood pressure, insulin resistance, body mass index (BMI) and lipid levels are explained by genetic components [13, 18–22]. Genome-wide association studies (GWAS) revealed that genes coding for adipokines or proteins implicated in lipoprotein metabolism and inflammation are linked to the pathogenesis of MetS [13]. Obesity is influenced by variants in genes regulating food intake, energy metabolism and neuroendocrine pathways [18, 23, 24]. Numerous genes regulating β-cells function and insulin secretion explain a significant fraction of insulin resistance [25, 26], while variants in genes related to lipoprotein metabolism could explain up to 70% of lipid level inheritance [22, 27–29].

Despite their importance, only a few studies evaluating the cardiometabolic risk of cALL survivors have taken genetic factors into consideration [30–32]. The identification of genetic biomarkers could help pinpoint high-risk individuals and develop prevention strategies to counter the development of late cardiometabolic complications. Even with the success of GWAS in identifying genetic predisposition, only 10% of the genetic variance of complex diseases can be explained by common variants [26, 33]. The missing genetic contribution might be attributed to rare variants that were not captured by traditional GWAS [34, 35] or to the combined impact of rare and common variants [36]. With next-generation sequencing technologies, it is now possible to have simultaneously access to both common and rare variants for genetic association studies [37]. The aim of this study was to assess the contribution of both rare and common genetic variants in the prevalence of cardiometabolic complication in a cohort of cALL survivors.

## Methods

### Cohort

Participants included were treated for cALL at Sainte-Justine University Health Center (SJUHC, Montreal, Canada) with the Dana Farber Cancer Institute (DFCI) protocols [38]. The cALL survivors were recruited as part of the PETALE study at SJUHC and had an average of 15.5 years (+/- 5.2 SD) after diagnosis [39]. Subjects who were less than 19 years old at diagnosis, more than 5 years post diagnosis, free of relapse, and who did not receive hematopoietic stem cell transplantation were invited to participate. To limit heterogeneity, the emphasis was put on pre-B ALL since this type is the most frequent [40, 41]. Participants were mainly of French Canadian origin [42, 43]. During their medical visits, participants were subjected to a series of genetic and biochemical analyses and examined by a multidisciplinary team of health professionals including physicians, nutritionists, physiotherapists and psychotherapists. The study was approved by the Institutional Review Board of SJUHC and investigations were carried out in accordance with the principles of the Declaration of Helsinki. Written informed consent was obtained from study participants or parents/guardians.

### Classification of cardiometabolic risk factors

The presence of the cardiometabolic risk factors, obesity, insulin resistance, dyslipidemia and pre-HTN was assessed in all subjects. In adults, obesity was defined as a BMI ≥30 kg/m^2^ and/or having a waist circumference ≥88 cm (women) or 102 cm (men) [44]. In children, BMI ≥97^th^ percentile according to the BMI charts of the World Health Organization [45] and/or waist circumference ≥95^th^ percentile defined obesity [46]. Blood pressure was measured on the right arm in the morning at rest. In adults, blood pressure ≥130/85 and <140/90 mmHg determined arterial pre-HTN and ≥140/90 mmHg HTN [47]. For children, we used current recommendations according to age and height: blood pressure ≥90^th^ and <95^th^ percentile indicated pre-HTN and ≥95^th^ percentile HTN [48, 49]. Elevated fasting glucose, glycated hemoglobin (HbA1c) and/or homeostasis model assessment (HOMA-IR) were used to identify insulin resistance. Cut-off values were fasting glucose ≥6.1 mmol/L [50] and HbA1c ≥6% [50] for both adults and children. HOMA-IR ≥2.86 (adults) [2, 51] and ≥95^th^ percentile for a pediatric reference population [52] were considered elevated. Dyslipidemia was defined based on high low-density lipoprotein-cholesterol (LDL-C), triglycerides (TG) and/or low high-density lipoprotein-cholesterol (HDL-C) concentrations. For adults, thresholds were LDL-C ≥3.4 mmol/L [53–55], TG ≥1.7mmol/L [53, 55, 56] and HDL-C <1.03 mmol/L in men and <1.3 in women [56]. For children, the values were compared to the National Heart, Lung and Blood Institute guidelines for age and gender [57]. Accumulation of cardiometabolic risk factors was determined by adding the presence of dyslipidemia, pre-HTN/HTN, insulin resistance and obesity. Participants with 3 or more risk factors were defined as “extreme phenotype” while those without risk factor were defined as “healthy”.

### Nutritional evaluation

Participants’ dietary intakes were collected using a validated interviewer-administered food frequency questionnaire (FFQ) [58] combined with a 3-day food record. Evaluation of nutrient intakes was performed using the Nutrition Data System for Research software v.4.03 [59]. A validated Mediterranean score calculated on a nine-point scale [60] was used to assess overall diet quality. Differences between calorie intake (calculated with the Institute of Medicine equations [61]) and estimated energy requirement (accounting for level of physical activity, equations shown in Table 1 [62]) determined energy balance.

**Table 1:** Estimated energy requirement equations

Group	Equation EER (kcal/d)
Boys 3-8 y	88.5 - (61.9 × age [y]) + PA × {(26.7 × weight [kg] + 903 × height [m])} + 20
Boys 9-18 y	88.5 - (61.9 × age [y]) + PA × {(26.7 × weight [kg] + 903 × height [m])} + 25
Men ≥19 y	662 - (9.53 × age [y]) + PA × {(15.91 × weight [kg]) + (539.6 × height [m])}
Girls 3-8 y	135.3 - (30.8 × age [y]) + PA × {(10.0 × weight [kg]) + (934 × height [m])} + 20
Girls 9-18 y	135.3 - (30.8 × age [y]) + PA × {(10.0 × weight [kg]) + (934 × height [m])} + 25
Women ≥19 y	354 - (6.91 × age [y]) + PA × {(9.36 × weight [kg]) + (726 × height [m])}

*PA* Physical activity coefficient, *y* years, *EER* estimated energy requirement

### Chemotherapeutic medication dose estimation

Theoretical cumulative doses of glucocorticoids (in prednisone equivalent [mg/m^2^]), methotrexate (mg/m^2^) and asparaginase (mg/m^2^) were calculated for each participant according to DFCI treatment protocols [38]. Exposure and doses of cranial radiotherapy were recorded according to protocol.

### Genetic data treatment and selection of variants

**Fig. 1:**
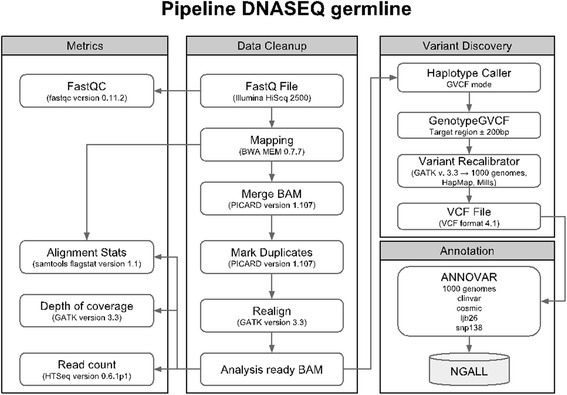
Germline variants analysis pipeline

We performed whole-exome sequencing (WES) on a total of 209 participants from the PETALE cohort. Sequencing data were obtained from SJUHC and Génome Québec Integrated Centre for Pediatric Clinical Genomic using the SOLiD (ThermoFisher Scientific) or Illumina HiSeq 2500 platforms and were aligned on the Hg19 reference genome (Fig. 1). Rare and common variants with a predicted functional impact on protein were identified by the functional annotation from ANNOVAR [63]. Only variants with a PolyPhen-2 score ≥0.85 [64] or a SIFT score ≤0.1 [65, 66] were labeled as “potentially damaging” and used for further analyses. Two lists were assembled; the first was composed of genes involved in methotrexate and corticoid metabolic pathways [67] and few genes of lipid metabolism shown to affect corticosteroid-related complications such as hypertension or osteonecrosis [68, 69]. The second list contained genes related to cardiometabolic pathways that were selected based on gene ontology terms using GOrilla [70, 71] and DisGeNET [72–75]. Variants were defined as rare (minor allele frequency (MAF) <5%) and common (MAF ≥5%) according to the reported frequency in the 1000genome [76] and ESP6500 [77] datasets for Caucasian populations. A total of 198 variants in the cardiometabolic list and 7 variants in the methotrexate and corticoid list did not conform to the Hardy-Weinberg equilibrium and were rejected.

### Power analysis

We used Quanto version 1.2.4 to compute power analysis at 80% [78] and Bonferroni correction for the number of SNPs or genes tested. The power analysis for common variant revealed that odds ratio (OR) ranging from 3 to 11 (depending on phenotype analyzed) for variants with MAF of 5-30% can be detected, whereas the lowest OR for rare variants, assuming a MAF of 0.01 that can be detected with a given sample size, was 16.

### Association studies and statistical analyses

Association between cardiometabolic risk factors and common variants were studied using PLINK (http://zzz.bwh.harvard.edu/plink/) [79, 80]. For each association, we also determined the genetic model in which the common variant affects the phenotype: dominant model (one variant allele impacts the phenotype), recessive model (two variant alleles are needed to modify the phenotype) and additive model (accumulation of variant alleles causes a gradation in the risk of developing the phenotype). Association analyses of rare variants were performed using the SKAT-O test in the SKAT package (https://cran.r-project.org/web/packages/SKAT/index.html) [35] developed for the open software R [81]. Combined rare and common variant analyses were also done with the SKAT package. The Benjamini and Hochberg method (FDR) was used to correct for multiple testing for each list and variants with a FDR less than 0.20 were kept for further analyses [81]. Selected polymorphisms were analyzed using a logistic regression model including eight covariables: age at interview, gender, cumulative doses of corticoids, methotrexate and asparaginase, exposure or not to cranial radiotherapy, Mediterranean diet score and energy balance. Finally, we used chi-square tests to compare the prevalence of cardiometabolic complications between children and adults. Statistical analyses were performed using SPSS version 22.0 [82].

## Results

### Cohort characteristics

The characteristics of the cohort are presented in Table 2. The cohort (53.6% female) was mostly composed of adolescents and young adults (median age of 22.4 years). Dyslipidemia was the most prevalent cardiometabolic risk factor (41.8%), followed by obesity (33.0%), insulin resistance (18.5%) and pre-HTN (10.1%). Dyslipidemia was the only risk factor for which we observed a significant difference between children and adults (30.2% vs. 46.9%, *P*<0.025). Of note, less than 40% of the cohort was classified as “healthy” (no MetS risk factor) and 10.7% as “extreme phenotype” (≥3 MetS risk factors).

**Table 2:** Characteristics of the PETALE cohort

	Total cohort	Adults	Children	*p*-value
Gender, n (%)				
Male	97 (46.4)	68 (46.6)	29 (46.0)	0.942
Female	112 (53.6)	78 (53.4)	34 (54.0)	
Age, median (range)	22.4 (8.5-41.0)	24.9 (18.1-41.0)	16.2 (8.5-17.9)	
Phenotype, n (%)				
Obesity	69 (33.0)	48 (32.9)	21 (33.3)	0.949
Pre-hypertension	21 (10.1)	16 (10.9)	5 (7.9)	0.505
Insulin resistance	38 (18.5)	29 (20.1)	9 (14.5)	0.34
Dyslipidemia	87 (41.8)	68 (46.9)	19 (30.2)	0.025
Extreme phenotype	22 (10.7)	18 (12.5)	4 (6.5)	0.197
Number of risk factors				
0	81 (39.3)	51 (35.4)	30 (48.4)	0.388
1	62 (30.1)	45 (31.3)	17 (27.4)	
2	41 (19.9)	30 (20.8)	11 (17.7)	
3	19 (9.2)	16 (11.1)	3 (4.9)	
4	3 (1.5)	2 (1.4)	1 (1.6)	

Extreme phenotype: Three and more cardiometabolic risk factor

Chi-square tests were used to compare the prevalence of cardiometabolic complications between children and adults

### Genetic associations with cardiometabolic candidate genes

**Fig. 2:**
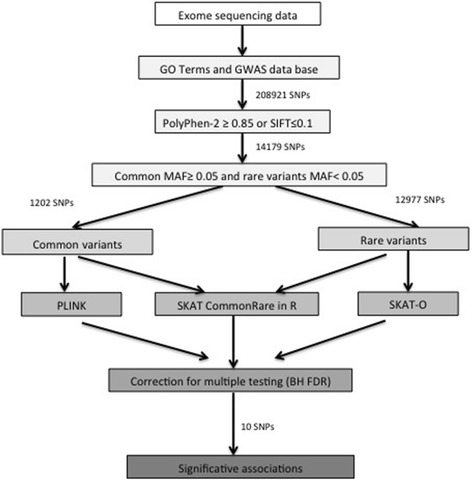
Processing of single nucleotide polymorphism for cardiometabolic candidate genes

We analyzed 1,202 common variants from the cardiometabolic candidate gene list (Fig. 2). We found associations between common variants and two phenotypes (Table 3): dyslipidemia and the extreme phenotype. Eukaryotic Translation Initiation Factor 4B (*EIF4B*) (FDR 0.18) and 2-oxoglutarate and iron dependent oxygenase domain containing 3 (*OGFOD3*) (FDR 0.18) was associated with dyslipidemia while extreme phenotype was linked to BCL2 Associated Agonist Of Cell Death (*BAD*) (FDR 0.20) and Fc Receptor Like 3 (*FCRL3*) (FDR 0.20). The SKAT-O test performed on the 12,977 rare variants did not reveal any significant association. The rare/common variant combined analysis showed associations between the extreme phenotype and 3 genes: *BAD* (FDR 0.09), *FCRL3* (FDR 0.09) and *EIF4B* (FDR 0.10) (Table 3).

**Table 3:** Significant genetic associations with cardiometabolic candidate genes

Common Variants							
	Gene	SNP ID		MAF	*p*-value	FDR	Model
Dyslipidemia	*EIF4B*	rs146008363		0.05	0.00018	0.180	DOM
	*OGFOD3*	rs62079523		0.33	0.00032	0.180	DOM
Extreme phenotype	*BAD*	rs2286615		0.10	0.00034	0.200	DOM
	*FCRL3*	rs2282284		0.03	0.00042	0.200	DOM
Common/Rare variants							
	Gene	Rare (n)	Common (n)	*p*-value	FDR		
Extreme phenotype	*BAD*	3	1	5.79x10^-5^	0.087		
	*FCRL3*	2	1	3.86x10^-5^	0.087		
	*EIF4B*	1	1	0.00010	0.100		

*MAF* Minor allele frequency, *DOM* Dominant effect, *Rare (n)* Number of rare variants analyzed in the gene, *Common (n)* Number of common variants analyzed in the gene, *Extreme phenotype* Three and more cardiometabolic risk factor

### Genetic associations with methotrexate and corticosteroid candidate genes

**Fig. 3:**
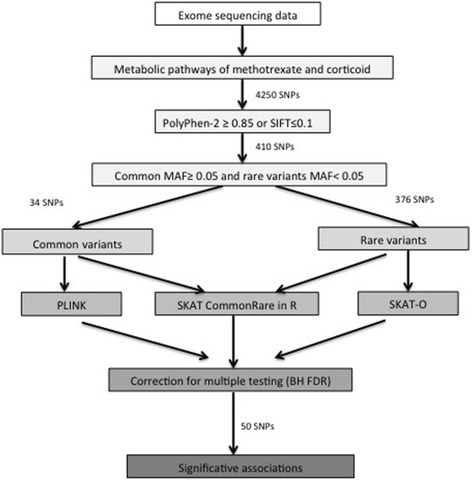
Processing of single nucleotide polymorphism for methotrexate and corticoid pathways’ candidate genes

Next, we studied 34 common variants in the methotrexate/corticoid candidate gene list (Fig. 3). For dyslipidemia, we observed associations with *BAD* (FDR 0.02) and Apolipoprotein B (*APOB*) (FDR 0.11) (Table 4). *BAD* was also associated with the extreme phenotype (FDR 0.009), insulin resistance (FDR 0.07) and obesity (FDR 0.08). Moreover, insulin resistance was associated with a common variant in Serpin Family A Member 6 (*SERPINA6*) (FDR 0.07) (Table 4). The SKAT-O analysis for 376 rare variants revealed associations between glucocorticoid receptor (Nuclear Receptor Subfamily 3 Group C Member 1, *NR3C1,* FDR 0.17) and the extreme phenotype as well as between pre-HTN and Corticotropin Releasing Hormone Receptor 1 (*CRHR1*) (FDR 0.20) and Corticotropin Releasing Hormone Receptor 2 (*CRHR2*) (FDR 0.20) (Table 4). Combined rare and common variant analyses exhibited 8 associations: *BAD* (FDR 0.04), *APOB* (FDR 0.12), Cystathionine-Beta-Synthase (*CBS*) (FDR 0.12) and Solute Carrier Organic Anion Transporter Family Member 4C1 (*SLCO4C1*) (FDR 0.14) with dyslipidemia; *BAD* (FDR 0.003) and *NR3C1* (FDR 0.15) with the extreme phenotype; and *CRHR1* (FDR 0.14) and *CRHR2* (FDR 0.14) with pre-HTN (Table 4).

**Table 4:** Significant genetic associations with methotrexate and corticosteroid candidate genes

Common Variants						
	Gene	SNP ID	MAF	*p*-value	FDR	Model
Dyslipidemia	*BAD*	rs2286615	0.10	0.00065	0.021	ADD
	*APOB*	rs676210	0.23	0.0069	0.110	DOM
Extreme phenotype	*BAD*	rs2286615	0.10	0.00034	0.0089	ADD, DOM
Insulin resistance	*BAD*	rs2286615	0.10	0.0044	0.069	DOM
	*SERPINA6*	rs2228541	0.50	0.0051	0.069	ADD, DOM, REC
Obesity	*BAD*	rs2286615	0.10	0.0025	0.081	ADD, DOM
Rare variants						
	Gene	Rare (n)		*p*-value	FDR	
Extreme phenotype	*NR3C1*	2		0.0021	0.17	
Pre-hypertension	*CRHR1*	1		0.0025	0.20	
	*CRHR2*	2		0.0048	0.20	
Common/Rare variants						
	Gene	Rare (n)	Common (n)	*p*-value	FDR	
Dyslipidemia	*BAD*	3	1	0.00049	0.040	
	*APOB*	30	3	0.0028	0.12	
	*CBS*	3	0	0.0042	0.12	
	*SLCO4C1*	4	0	0.0066	0.14	
Extreme phenotype	*BAD*	3	1	3.35x10^-5^	0.0028	
	*NR3C1*	2	0	0.0037	0.15	
Pre-hypertension	*CRHR1*	1	0	0.0032	0.14	
	*CRHR2*	2	0	0.0033	0.14	

*MAF* Minor allele frequency, *DOM* Dominant effect, *ADD* Additive effect, *REC* Recessive effect, *Rare (n)* Number of rare variants analyzed in the gene, *Common (n)* Number of common variants analyzed in the gene, *Extreme phenotype* Three and more cardiometabolic risk factor

### Logistic regression analysis with significant cardiometabolic candidate genes

Significant genetic variants were further analyzed in a logistic regression model including 8 covariables (see Methods). Analysis revealed independent associations between the extreme phenotype and the common variant rs2286615 in *BAD* (*p*=0.006, in a dominant effect model), age at interview (*p*=0.04), and exposure to cranial radiotherapy (*p*=0.04) (Table 5). The common and rare variant analysis showed associations between the extreme phenotype and age (*p*=0.03), cumulative doses of methotrexate (*p*=0.05), exposure to cranial radiotherapy (*p*=0.04) and the *BAD* gene (*p*=0.003) (Table 5). The common variant rs2282284 in *FCRL3* was also associated with the extreme phenotype with a dominant effect (*p*=0.006) (Table 5). *FCRL3* (rare and common variants) was associated with the extreme phenotype (*p*=0.04) while no other covariable reached statistical significance in this model (Table 5). The variant rs62079523 in *OGFOD3*, associated with dyslipidemia in the dominant model, was found highly significant in the logistic regression model (*p*=0.005) (Table 5).

**Table 5:** Logistic regression model with significant cardiometabolic candidate genes

	Extreme Phenotype				Dyslipidemia
	*BAD*/rs2286615 (C, DOM)	*FCRL3*/rs2282284 (C,DOM)	*BAD* (CR)	*FCRL3* (CR)	*OGFOD3/rs62079523 (C, DOM)*
	OR (95% CI) *p*-value				
Age	1.219 (1.005-1.478)	1.151 (0.993-1.334)	1.213 (1.017-1.447)	1.150 (0.993-1.332)	1.033 (0.962-1.109)
	**0.044**	0.062	**0.032**	0.062	0.374
Gender	1.152 (0.216-6.142)	1.062 (0.268-4.201)	1.624 (0.340-7.749)	1.039 (0.266-4.063)	0.720 (0.360-1.439)
	0.869	0.932	0.543	0.956	0.352
Corticoid	1.000 (1.000-1.000)	1.000 (1.000-1.000)	1.000 (1.000-1.000)	1.000 (1.000-1.000)	1.000 (1.000-1.000)
	0.577	0.570	0.355	0.574	0.528
Asparaginase	1.000 (1.000-1.000)	1.000 (1.000-1.000)	1.000 (1.000-1.000)	1.000 (1.000-1.000)	1.000 (1.000-1.000)
	0.714	0.158	0.444	0.270	0.346
Methotrexate	0.999 (0.999-1.000)	1.000 (0.999-1.000)	0.999 (0.999-1.000)	1.000 (0.999-1.000)	1.000 (1.000-1.000)
	0.075	0.800	**0.048**	0.729	0.696
CRT	14.506 (1.116-188.530)	4.938 (0.687-35.491)	16.098 (1.220-212.463)	3.544 (0.561-22.385)	1.708 (0.668-4.366)
	**0.041**	0.112	**0.035**	0.178	0.264
Energy balance	0.999 (0.998-1.001)	0.999 (0.998-1.000)	1.000 (0.998-1.001)	0.999 (0.999-1.000)	1.000 (0.999-1.000)
	0.297	0.304	0.421	0.306	0.210
Med score	0.652 (0.319-1.329)	0.884 (0.518-1.509)	0.752 (0.374-1.513)	0.815 (0.491-1.353)	1.008 (0.807-1.259)
	0.239	0.651	0.425	0.430	0.944
SNP	57.900 (3.152-1063.462)	67.983 (3.393-1362.288)	68.819 (4.202-1159.995)	11.695 (1.150-118.907)	2.712 (1.352-5.442)
	**0.006**	**0.006**	**0.003**	**0.038**	**0.005**

Top: Odds ratio [95% CI], bottom: *p*-value

Boldface: significant association

*C* common, *CR* common/rare, *DOM* Dominant effect, *CRT* Cranial radiotherapy, *Med score* Mediterranean diet score, *Extreme phenotype* Three and more cardiometabolic risk factor

### Logistic regression model with significant methotrexate and corticoid candidate genes

The results of the logistic regression analyses for the significant genes in the methotrexate/corticosteroid list are presented in Table 6. We found that the common *BAD* variant rs2286615 was associated with the extreme phenotype (*p*=0.006) in a dominant and additive effect as it was with age (*p*=0.04) and cranial radiotherapy (*p*=0.04). The combined analysis of common and rare *BAD* variants was significant for the extreme phenotype (*p*=0.003). In this model, age (*p*=0.03), cumulative doses of methotrexate (*p*=0.05) and cranial radiotherapy (*p*=0.04) were also significant. *BAD* was associated with dyslipidemia for the common variant rs2286615 (*p*=0.008, additive model) and for the common and rare variants (*p*=0.006). Also the rs2286615 variant was associated in dominant (*p*=0.009) and additive (*p*=0.006) effect model with the presence of obesity. Rs676210, a variant in *APOB*, had a dominant effect on the risk of dyslipidemia and was the only significant association in the logistic regression model (*p*=0.02). An additive effect was observed for the common variant rs2228541 (*SERPINA6*) and insulin resistance (*p*=0.05). Finally, the logistic regression model including rare variants in *CRHR1* and *CRHR2* for pre-HTN revealed associations for gender (*p*=0.03) but the genetic associations did not reach statistical significance.

**Table 6:** Logistic regression model with significant methotrexate and corticoid candidate genes

	Extreme phenotype		Dyslipidemia		
	*BAD*/rs2286615 (C, DOM, ADD)	*BAD* (CR)	*APOB*/rs676210 (C, DOM)	*BAD*/rs2286615 (C, ADD)	*BAD* (CR)
	OR (95% CI) *p*-value				
Age	1.219 (1.005-1.478)	1.213 (1.017-1.447)	1.041 (0.971-1.117)	1.047 (0.961-1.141)	1.037 (0.966-1.114)
	**0.044**	**0.032**	0.259	0.292	0.313
Gender	1.152 (0.216-6.142)	1.624 (0.340-7.749)	0.726 (0.365-1.444)	1.058 (0.453-2.472)	1.043 (0.506-2.151)
	0.869	0.543	0.361	0.896	0.908
Corticoid	1.000 (1.000-1.000)	1.000 (1.000-1.000)	1.000 (1.000-1.000)	1.000 (1.000-1.000)	1.000 (1.000-1.000)
	0.577	0.355	0.411	0.519	0.571
Asparaginase	1.000 (1.000-1.000)	1.000 (1.000-1.000)	1.000 (1.000-1.000)	1.000 (1.000-1.000)	1.000 (1.000-1.000)
	0.714	0.444	0.372	0.704	0.344
Methotrexate	0.999 (0.999-1.000)	0.999 (0.999-1.000)	1.000 (1.000-1.000)	1.000 (1.000-1.000)	1.000 (1.000-1.000)
	0.075	**0.048**	0.783	0.225	0.642
CRT	14.506 (1.116-188.530)	16.098 (1.220-212.463)	1.572 (0.619-3.994)	2.361 (0.751-7.425)	2.255 (0.843-6.033)
	0.041	0.035	0.341	0.142	0.105
Energy balance	0.999 (0.998-1.001)	1.000 (0.998-1.001)	1.000 (0.999-1.000)	1.000 (0.999-1.000)	1.000 (0.999-1.000)
	0.297	0.421	0.469	0.319	0.350
Med score	0.652 (0.319-1.329)	0.752 (0.374-1.513)	1.017 (0.813-1.272)	1.029 (0.793-1.334)	0.984 (0.780-1.240)
	0.239	0.425	0.885	0.831	0.888
SNP	57.900 (3.152-1063.462)	69.819 (4.202-1159.995)	0.434 (0.215-0.877)	4.022 (1.441-11.226)	3.560 (1.427-8.882)
	**0.006**	**0.003**	**0.020**	**0.008**	**0.006**
	Obesity		Pre-hypertension		Insulin resistance
	*BAD*/rs2286615 (C, DOM)	*BAD*/rs2286615 (C, ADD)	*CRHR1* (R)	*CRHR2* (R)	*SERPINA6*/rs2228541 (C, ADD)
	OR (95% CI) *p*-value				
Age	0.996 (0.914-1.085)	0.998 (0.916-1.087)	1.010 (0.873-1.169)	0.995 (0.869-1.139)	1.085 (0.993-1.185)
	0.926	0.959	0.896	0.940	0.073
Gender	1.979 (0.824-4.750)	2.073 (0.854-5.034)	0.081 (0.009-0.741)	0.165 (0.033-0.809)	1.330 (0.562-3.150)
	0.127	0.107	**0.026**	**0.026**	0.517
Corticoid	1.000 (1.000-1.000)	1.000 (1.000-1.000)	1.000 (1.000-1.000)	1.000 (1.000-1.000)	1.000 (1.000-1.000)
	0.998	0.971	0.828	0.830	0.898
Asparaginase	1.000 (1.000-1.000)	1.000 (1.000-1.000)	1.000 (1.000-1.000)	1.000 (1.000-1.000)	1.000 (1.000-1.000)
	0.863	0.824	0.621	0.452	0.141
Methotrexate	1.000 (1.000-1.000)	1.000 (1.000-1.000)	1.000 (0.999-1.000)	1.000 (0.999-1.000)	1.000 (1.000-1.000)
	0.825	0.786	0.160	0.364	0.935
CRT	1.915 (0.607-6.038)	2.029 (0.636-6.480)	7.685 (0.713-82.885)	3.558 (0.550-23.009)	1.539 (0.463-5.118)
	0.267	0.232	0.093	0.183	0.482
Energy balance	1.000 (0.999-1.000)	1.000 (0.999-1.000)	1.000 (0.999-1.001)	0.999 (0.998-1.000)	1.000 (0.999-1.000)
	0.137	0.153	0.573	0.168	0.257
Med score	0.921 (0.710-1.195)	0.911 (0.700-1.184)	0.871 (0.562-1.349)	0.806 (0.555-1.170)	0.939 (0.713-1.236)
	0.534	0.485	0.536	0.257	0.652
SNP	3.993 (1.410-11.307)	4.044 (1.504-10.879)	76.406 (0.948-6158.616)	3.417 (0.384-30.405)	0.534 (0.286-0.998)
	**0.009**	**0.006**	0.053	0.271	**0.049**

Top: Odds ratio (95% CI), bottom: *p*-value

Boldface: Significant association

*C* common, *CR* common/rare, *R* rare, *DOM* Dominant effect, *ADD* Additive effect, *CRT* Cranial radiotherapy, *Med score* Mediterranean diet score, *Extreme phenotype* Three and more cardiometabolic risk factor

## Discussion

This study is among the first studies to address the contribution of genetic determinants in the development of long-term cardiometabolic complications in cALL survivors. Globally, we found that the development of an extreme cardiometabolic phenotype can be predicted by common and rare variants in *BAD* and *FCRL3*. The presence of dyslipidemia in cALL survivors is influenced by common variants in *OGFOD3* and *APOB* and by common and rare variants in *BAD*. Obesity was predicted by a common variant in *BAD* and insulin resistance was associated with a common variant in *SERPINA6*. Pre-HTN was related to survivors’ gender as being a female was found protective for this complication. This gender difference between men and women before menopause has been well described in the literature [83, 84].

We found similar prevalence of obesity in children and in adults, suggesting that obesity acquired during childhood following the treatments persists thorough adulthood, a hypothesis supported by other studies [85–87]. Obesity is central to the MetS and is a major risk factor for HTN, dyslipidemia and insulin resistance [23, 88]. The PETALE cohort appeared to be particularly affected by dyslipidemia as almost 47% of adults were afflicted. For comparison, a study conducted in a population of young Canadian adults (18-39 years old) revealed that 34% were affected by dyslipidemia [89]. Given their young age, this finding raises concerns for the long-term cardiovascular risk of cALL survivors. In fact, 60% of our cohort was affected by at least one cardiometabolic risk factor, 10.7% of them being classified as extreme phenotypes. The observation related to the median age of 22.4 years places the survivors at high risk for early cardiovascular disease.

The common variant rs2286615 in the *BAD* gene was associated with extreme phenotype and obesity, whereas interactions between rare and common variants were linked to extreme phenotype and dyslipidemia. *BAD* is a gene that codes for a protein member of the pro-apoptotic Bcl-2 protein family named "Bcl2-associated agonist of cell death". In response to activation by hypoxia, reactive oxygen species, nutrient withdrawal or DNA damage, the pro-apoptotic proteins in the Bcl-2 family create pores in the mitochondrial membrane by which cytochrome can be released, triggering the apoptotic cascade leading to cell death [90]. *BAD* could have an impact on the development of insulin resistance since an imbalance between pro-apoptotic and anti-apoptotic proteins in situation of high blood glucose promotes β-cell apoptosis [90], the latest playing an important role in the pathophysiology of type 2 diabetes [90]. Studies suggest that *BAD* has a role in β-cell function and can promote glucose-stimulated insulin secretion [91–93]. Besides, it has been reported that *BAD* suppresses the formation of tumors in lymphocytes and that *Bad*-deficient mice are at higher risk of lymphoma and leukemia [94]. In another study, *Bad*-deficient mice were prone to cancer and did not respond adequately to DNA damage [95]. This gene is thus a suitable candidate to explain a common etiology between the predisposition to cardiometabolic complication and hematologic malignancies. Because *BAD* is recurrent in almost all associations with the cardiometabolic risk factors in our study, we can conclude that it is a strong candidate gene for MetS in cALL survivors. It is possible that through its effects on insulin resistance, *BAD* can predispose the participants to develop obesity, dyslipidemia and pre-HTN [8, 96–98]. As expected, age had an impact on the presence of the extreme phenotype in the model with *BAD*. We observed that adults were more affected by cardiometabolic complications than children. This can be explained by the fact that the establishment of cardiometabolic risk factors is a long-term and latent process. Other studies on cALL survivors have reported that obesity, diabetes and the metabolic syndrome are more frequent in patients who received cranial radiotherapy [9, 10, 99]. This is in accordance with our results showing that cranial radiotherapy significantly increased the risk of extreme phenotype. This could be caused by the impact of radiotherapy on the brain satiety control center and on hormones implicated in energy regulation [1, 100, 101]. Indeed, damages caused by cranial radiotherapy could lead to growth hormone deficiency and then to the development of metabolic disorders such as visceral obesity, hyperinsulinemia and low HDL-C [102].

Carriers of one allele of the variant rs2282284 in *FCRL3*, encoding for a protein that is part of the immunoglobulin receptors, were at increased risk of presenting the extreme phenotype. The common and rare variant analysis also revealed a significant association between *FCRL3* and the extreme phenotype. It has a role in immune function and is expressed in secondary lymphoid organs, mostly in B lymphocytes [103]. This gene has been linked to rheumatoid arthritis, autoimmune thyroid disease and systemic lupus erythematosus [103–105]. In particular, the SNP rs2282284 has been associated to higher risk of neuromyelitis optica (a severe inflammatory demyelinating disease of the central nervous system) [106] and correlated with the risk of multiple sclerosis [107] in the Chinese Han population. *FCRL3* role in immune regulation is of interest given the contribution of inflammation in MetS pathogenesis [7, 108, 109].

The common variant rs62079523 in *OGFOD3* was found associated with dyslipidemia in the dominant model. No clear function has been reported for this gene in the literature but it was linked with the gene ontology term 2-oxoglutarate and iron-dependent oxygenase domain-containing protein 3 in our analysis.

We found the common variant rs676210 in *APOB* correlated with the development of dyslipidemia, the presence of the minor allele (A) being protective for the outcome. *APOB* codes for the apolipoproteins B-48 and B-100 that play a central role in lipid transport and metabolism. They are the main apolipoproteins of chylomicron, very low density lipoprotein (VLDL) and LDL [110, 111]. The rs676210 polymorphism induces a change (proline to leucine) in position 2739 of the protein, thereby not affecting apolipoprotein B-48, a 2152 amino acid protein that is the result of *APOB* RNA editing [112, 113]. In line with our results, it was demonstrated that the carriers of the major allele (G) had higher levels of oxidized LDL [114, 115] that predispose to atherosclerosis. However, these studies failed to find an association between the SNP and risk of cardiovascular events [114]. Moreover, in comparison with the carriers of the major allele G, the minor allele A was linked to lower TG, total cholesterol and LDL-C levels and with higher HDL-C [114]. This profile is favorable to a healthy cardiovascular system [114] and is in agreement with our findings. A study also reported a higher prevalence of glucocorticoid-induced hypertension in patients with an *APOB* polymorphism [68], which demonstrate the multiple impacts this gene can have on cardiovascular health.

The variant rs2228541 in *SERPINA6* was associated with a decreased risk of insulin resistance. Similarly, common variants at the *SERPINA6* locus were found associated with plasma levels of cortisol in a study comprising of 12,597 Caucasians [116]. It was postulated that this effect was mediated by changes in the total cortisol binding capacity by the corticosteroid binding globulin. Variations in plasma cortisol levels have been associated with cardiovascular disease, obesity, type 2 diabetes, HTN and dyslipidemia [116]. Thus, this SNP could be linked to cortisol levels and thus predisposes to type 2 diabetes. However, because data was not available, we could not determine if *SERPINA6* variants were associated with the development of hyperglycemia during ALL treatment.

Rare variants in the *CRHR1* and *CRHR2* genes were linked to pre-HTN. This effect was lost in the logistic regression model, but the latter uncovered the impact of gender on the phenotype, women being protective for the outcome. The unequal distribution of the phenotype between the genders (17.53% in men and 3.57% in women) could probably explain the observed relationship.

On the other hand, corticoid and asparaginase cumulative doses did not have a significant impact on the development of cardiometabolic risk factors in our study. It appeared that exposure to cranial radiotherapy was the major risk factor to predict the development of late cardiometabolic complications. Moreover, neither the quality of diet (evaluated with the Mediterranean diet score) nor the excess in calories were found significantly associated with the outcomes in our models.

Standard contingency tables and regression model allowed us to study common variants but did not provide enough power to study rare variants [36]. We had to use a technique that analyzes the cumulative effects of different rare variants on the same gene [117]. We also performed combined rare and common variants analysis in order to detect interactions. With this strategy we were able to discover associations that could not be seen with traditional associations studies, consisting the strength of this study. The limited sample size did not provide us with optimal power, especially for rare variants analysis. Replication studies in other cohorts of cALL survivors will be needed to confirm the observed associations.

## Conclusions

This study contributes to better understand the genetic determinants in the development of long-term cardiometabolic complication in childhood ALL survivors. Genetic information associated with both common and rare variants can help predict the development of late onset cardiometabolic complications. Genetic biomarkers can be used to propose prevention strategies, personalize the treatment and the follow-up to minimize the long-term sequelae and increase the quality of life of this high-risk population.

## Abbreviations

BMI: Body mass index
cALL: Childhood acute lymphoblastic leukemia
FDR: False discovery rate
FFQ: Food frequency questionnaire
GWAS: Genome-wide association study
HbA1c: Glycated hemoglobin
HDL-C: High-density lipoprotein cholesterol
HOMA-IR: Homeostasis model assessment
HTN: Hypertension
LDL-C: Low-density lipoprotein cholesterol
MetS: Metabolic syndrome
PETALE: Prévenir les effets tardifs des traitements de la leucémie aigüe lymphoblastique chez l’enfant
SNP: Single nucleotide polymorphism
TG: Triglycerides
VLDL: Very low-density lipoprotein
WES: Whole exome sequencing

## References

[1] Ness KK, Armenian SH, Kadan-Lottick N, Gurney JG (2011). Adverse effects of treatment in childhood acute lymphoblastic leukemia: general overview and implications for long-term cardiac health. Expert Rev Hematol.

[2] Nottage KA, Ness KK, Li C, Srivastava D, Robison LL, Hudson MM (2014). Metabolic syndrome and cardiovascular risk among long-term survivors of acute lymphoblastic leukaemia - From the St. Jude Lifetime Cohort. Br J Haematol.

[3] Oeffinger KC, Mertens AC, Sklar CA, Kawashima T, Hudson MM, Meadows AT, Friedman DL, Marina N, Hobbie W, Kadan-Lottick NS (2006). Chronic health conditions in adult survivors of childhood cancer. N Engl J Med.

[4] Oeffinger KC, Buchanan GR, Eshelman DA, Denke MA, Andrews TC, Germak JA, Tomlinson GE, Snell LE, Foster BM (2001). Cardiovascular risk factors in young adult survivors of childhood acute lymphoblastic leukemia. J Pediatr Hematol Oncol.

[5] Neville KA, Cohn RJ, Steinbeck KS, Johnston K, Walker JL (2006). Hyperinsulinemia, impaired glucose tolerance, and diabetes mellitus in survivors of childhood cancer: prevalence and risk factors. J Clin Endocrinol Metab.

[6] Mudi A, Levy CS, Geel JA, Poole JE. Paediatric cancer survivors demonstrate a high rate of subclinical renal dysfunction. Pediatr Blood Cancer. 2016; 10.1002/pbc.26132 27393905

[7] Gardner GD, Shoback D. Obesity. In: Lange, editor. Greenspan : Greenspan Basic and Clinical Endocrinology. 9th ed. San Francisco: McGraw Hill. p. 2011.

[8] Longo LD, Kasper LD, Jameson L, Fauci AS, Hauser LS, Loscalzo J, et al. The Metabolic Syndrome. In: Harrison's Principles of Internal Medicine. 18th edition ed: McGraw Hill; 2012.

[9] Esbenshade AJ, Simmons JH, Koyama T, Koehler E, Whitlock JA, Friedman DL (2011). Body mass index and blood pressure changes over the course of treatment of pediatric acute lymphoblastic leukemia. Pediatr Blood Cancer.

[10] Marcoux S, Langlois-Pelletier C, Charpentier A-M, Laverdière C (2013). Prise en charge médicale des patients adultes ayant été soignés pour un cancer pédiatrique. MedActuel DPC.

[11] Navarro-Millan I, Charles-Schoeman C, Yang S, Bathon JM, Bridges SL, Chen L, Cofield SS, Dell'Italia LJ, Moreland LW, O'Dell JR (2013). Changes in lipoproteins associated with methotrexate or combination therapy in early rheumatoid arthritis: results from the treatment of early rheumatoid arthritis trial. Arthritis Rheum.

[12] Oeffinger KC (2008). Are survivors of acute lymphoblastic leukemia (ALL) at increased risk of cardiovascular disease?. Pediatr Blood Cancer.

[13] Aguilera CM, Olza J, Gil A (2013). Genetic susceptibility to obesity and metabolic syndrome in childhood. Nutricion hospitalaria.

[14] Carruba G, Cocciadiferro L, Di Cristina A, Granata OM, Dolcemascolo C, Campisi I, Zarcone M, Cinquegrani M, Traina A (2016). Nutrition, aging and cancer: lessons from dietary intervention studies. Immun Ageing.

[15] Schwingshackl L, Hoffmann G (2016). Does a Mediterranean-Type Diet Reduce Cancer Risk?. Current nutrition reports.

[16] Smith WA, Li C, Nottage KA, Mulrooney DA, Armstrong GT, Lanctot JQ, Chemaitilly W, Laver JH, Srivastava DK, Robison LL (2014). Lifestyle and metabolic syndrome in adult survivors of childhood cancer: a report from the St. Jude Lifetime Cohort Study. Cancer.

[17] Tonorezos ES, Robien K, Eshelman-Kent D, Moskowitz CS, Church TS, Ross R, Oeffinger KC (2013). Contribution of diet and physical activity to metabolic parameters among survivors of childhood leukemia. Cancer Causes Control.

[18] Speliotes EK, Willer CJ, Berndt SI, Monda KL, Thorleifsson G, Jackson AU, Lango Allen H, Lindgren CM, Luan J, Magi R (2010). Association analyses of 249,796 individuals reveal 18 new loci associated with body mass index. Nat Genet.

[19] Heller DA, de Faire U, Pedersen NL, Dahlen G, McClearn GE (1993). Genetic and environmental influences on serum lipid levels in twins. N Engl J Med.

[20] Brown AE, Walker M (2016). Genetics of Insulin Resistance and the Metabolic Syndrome. Curr Cardiol Rep.

[21] Goode EL, Cherny SS, Christian JC, Jarvik GP, de Andrade M (2007). Heritability of longitudinal measures of body mass index and lipid and lipoprotein levels in aging twins. Twin Res Hum Genet.

[22] Willer CJ, Sanna S, Jackson AU, Scuteri A, Bonnycastle LL, Clarke R, Heath SC, Timpson NJ, Najjar SS, Stringham HM (2008). Newly identified loci that influence lipid concentrations and risk of coronary artery disease. Nat Genet.

[23] Thorleifsson G, Walters GB, Gudbjartsson DF, Steinthorsdottir V, Sulem P, Helgadottir A, Styrkarsdottir U, Gretarsdottir S, Thorlacius S, Jonsdottir I (2009). Genome-wide association yields new sequence variants at seven loci that associate with measures of obesity. Nat Genet.

[24] Willer CJ, Speliotes EK, Loos RJ, Li S, Lindgren CM, Heid IM, Berndt SI, Elliott AL, Jackson AU, Lamina C (2009). Six new loci associated with body mass index highlight a neuronal influence on body weight regulation. Nat Genet.

[25] Genome-wide association study of 14,000 cases of seven common diseases and 3,000 shared controls. Nature. 2007;447(7145):661–78. 10.1038/nature05911 PMC2719288 17554300

[26] Frazer KA, Murray SS, Schork NJ, Topol EJ (2009). Human genetic variation and its contribution to complex traits. Nat Rev Genet.

[27] Kathiresan S, Melander O, Guiducci C, Surti A, Burtt NP, Rieder MJ, Cooper GM, Roos C, Voight BF, Havulinna AS (2008). Six new loci associated with blood low-density lipoprotein cholesterol, high-density lipoprotein cholesterol or triglycerides in humans. Nat Genet.

[28] Sandhu MS, Waterworth DM, Debenham SL, Wheeler E, Papadakis K, Zhao JH, Song K, Yuan X, Johnson T, Ashford S (2008). LDL-cholesterol concentrations: a genome-wide association study. Lancet.

[29] Turner SD, Berg RL, Linneman JG, Peissig PL, Crawford DC, Denny JC, Roden DM, McCarty CA, Ritchie MD, Wilke RA (2011). Knowledge-driven multi-locus analysis reveals gene-gene interactions influencing HDL cholesterol level in two independent EMR-linked biobanks. PLoS One.

[30] Ross JA, Oeffinger KC, Davies SM, Mertens AC, Langer EK, Kiffmeyer WR, Sklar CA, Stovall M, Yasui Y, Robison LL (2004). Genetic variation in the leptin receptor gene and obesity in survivors of childhood acute lymphoblastic leukemia: a report from the Childhood Cancer Survivor Study. J Clin Oncol.

[31] Szymon S, Bik-Multanowski M, Balwierz W, Pietrzyk JJ, Surmiak M, Strojny W, Galicka-Latala D, Gozdzik J (2011). Homozygosity for the rs9939609T allele of the FTO gene may have protective effect on becoming overweight in survivors of childhood acute lymphoblastic leukaemia. J Genet.

[32] Wilson CL, Liu W, Yang JJ, Kang G, Ojha RP, Neale GA, Srivastava DK, Gurney JG, Hudson MM, Robison LL (2015). Genetic and clinical factors associated with obesity among adult survivors of childhood cancer: A report from the St. Jude Lifetime Cohort. Cancer.

[33] Dering C, Hemmelmann C, Pugh E, Ziegler A (2011). Statistical analysis of rare sequence variants: an overview of collapsing methods. Genet Epidemiol.

[34] Manolio TA, Collins FS, Cox NJ, Goldstein DB, Hindorff LA, Hunter DJ, McCarthy MI, Ramos EM, Cardon LR, Chakravarti A (2009). Finding the missing heritability of complex diseases. Nature.

[35] MC W, Lee S, Cai T, Li Y, Boehnke M, Lin X (2011). Rare-variant association testing for sequencing data with the sequence kernel association test. Am J Hum Genet.

[36] Bansal V, Libiger O, Torkamani A, Schork NJ (2010). Statistical analysis strategies for association studies involving rare variants. Nat Rev Genet.

[37] Shendure J, Ji H (2008). Next-generation DNA sequencing. Nat Biotechnol.

[38] Silverman LB, Stevenson KE, O'Brien JE, Asselin BL, Barr RD, Clavell L, Cole PD, Kelly KM, Laverdiere C, Michon B (2010). Long-term results of Dana-Farber Cancer Institute ALL Consortium protocols for children with newly diagnosed acute lymphoblastic leukemia (1985-2000). Leukemia.

[39] Marcoux S, Drouin S, Laverdiere C, Alos N, Andelfinger GU, Bertout L, Curnier D, Friedrich MG, Kritikou EA, Lefebvre G, et al. The PETALE study: Late adverse effects and biomarkers in childhood acute lymphoblastic leukemia survivors. Pediatr Blood Cancer. 2016; 10.1002/pbc.26361 27917589

[40] Kumar V, Abbas AK, Fausto N, Aster CJ. Diseases of White Blood Cells, Lymph Nodes, Spleen and Thymus. In: Robbins and Cotran Pathologic Basis of Disease. 8th edition ed. Saunders: Elsevier. p. 2010.

[41] Onciu M (2009). Acute lymphoblastic leukemia. Hematology/oncology clinics of North America.

[42] Krajinovic M, Labuda D, Richer C, Karimi S, Sinnett D (1999). Susceptibility to childhood acute lymphoblastic leukemia: influence of CYP1A1, CYP2D6, GSTM1, and GSTT1 genetic polymorphisms. Blood.

[43] Labuda D, Zietkiewicz E, Labuda M (1997). The genetic clock and the age of the founder effect in growing populations: a lesson from French Canadians and Ashkenazim. Am J Hum Genet.

[44] IDF worldwide definition of the metabolic syndrome [http://www.idf.org/metabolic-syndrome]

[45] Growth references 5-19 years [http://www.who.int/growthref/who2007_bmi_for_age/en/]

[46] Katzmarzyk PT (2004). Waist circumference percentiles for Canadian youth 11-18y of age. Eur J Clin Nutr.

[47] CHEP [https://www.hypertension.ca/fr/chep]

[48] The fourth report on the diagnosis, evaluation, and treatment of high blood pressure in children and adolescents. Pediatrics. 2004;114(2 Suppl 4th Report):555–76. 15286277

[49] Growth Charts [http://www.cdc.gov/growthcharts]

[50] Cheng AY (2013). Canadian Diabetes Association 2013 clinical practice guidelines for the prevention and management of diabetes in Canada. Introduction. Can J Diabetes.

[51] Chen J, Wildman RP, Hamm LL, Muntner P, Reynolds K, Whelton PK, He J, Third National H, Nutrition Examination S (2004). Association between inflammation and insulin resistance in U.S. nondiabetic adults: results from the Third National Health and Nutrition Examination Survey. Diabetes care.

[52] Allard P, Delvin EE, Paradis G, Hanley JA, O'Loughlin J, Lavallee C, Levy E, Lambert M (2003). Distribution of fasting plasma insulin, free fatty acids, and glucose concentrations and of homeostasis model assessment of insulin resistance in a representative sample of Quebec children and adolescents. Clinical chemistry.

[53] High Cholesterol [https://www.mayoclinic.org/diseases-conditions/high-blood-cholesterol/diagnosis-treatment/drc-20350806]

[54] Executive Summary of The Third Report of The National Cholesterol Education Program (NCEP) Expert Panel on Detection, Evaluation, And Treatment of High Blood Cholesterol In Adults (Adult Treatment Panel III). JAMA. 2001;285(19):2486–97. 10.1001/jama.285.19.2486 11368702

[55] An International Atherosclerosis Society position paper: global recommendations for the management of dyslipidemia: executive summary. Atherosclerosis. 2014;232(2):410–3. 10.1016/j.atherosclerosis.2013.11.031 24468156

[56] Genest J, McPherson R, Frohlich J, Anderson T, Campbell N, Carpentier A, Couture P, Dufour R, Fodor G, Francis GA (2009). 2009 Canadian Cardiovascular Society/Canadian guidelines for the diagnosis and treatment of dyslipidemia and prevention of cardiovascular disease in the adult - 2009 recommendations. Can J Cardiol.

[57] Integrated Guidelines for Cardiovascular Health and Risk Reduction in Children and Adolescents [http://www.nhlbi.nih.gov/health-pro/guidelines/current/cardiovascular-health-pediatric-guidelines/]

[58] Goulet J, Nadeau G, Lapointe A, Lamarche B, Lemieux S (2004). Validity and reproducibility of an interviewer-administered food frequency questionnaire for healthy French-Canadian men and women. Nutrition journal.

[59] Schakel SF, Sievert YA, Buzzard IM (1988). Sources of data for developing and maintaining a nutrient database. J Am Diet Assoc.

[60] Martinez-Gonzalez MA, Fernandez-Jarne E, Serrano-Martinez M, Wright M, Gomez-Gracia E (2004). Development of a short dietary intake questionnaire for the quantitative estimation of adherence to a cardioprotective Mediterranean diet. Eur J Clin Nutr.

[61] Io M (2005). Dietary Reference Intakes for Energy, Carbohydrate, Fiber, Fat, Fatty Acids, Cholesterol, Protein, and Amino Acids (Macronutrients).

[62] Dietary Reference Intakes Tables [http://www.hc-sc.gc.ca]

[63] Wang K, Li M, Hakonarson H (2010). ANNOVAR: functional annotation of genetic variants from high-throughput sequencing data. Nucleic Acids Res.

[64] Adzhubei IA, Schmidt S, Peshkin L, Ramensky VE, Gerasimova A, Bork P, Kondrashov AS, Sunyaev SR (2010). A method and server for predicting damaging missense mutations. Nature methods.

[65] SIFT [http://sift.jcvi.org/]

[66] Ng PC, Henikoff S (2003). SIFT: predicting amino acid changes that affect protein function. Nucleic Acids Res.

[67] Whirl-Carrillo M, McDonagh EM, Hebert JM, Gong L, Sangkuhl K, Thorn CF, Altman RB, Klein TE (2012). Pharmacogenomics knowledge for personalized medicine. Clin Pharmacol Ther.

[68] Kamdem LK, Hamilton L, Cheng C, Liu W, Yang W, Johnson JA, Pui CH, Relling MV (2008). Genetic predictors of glucocorticoid-induced hypertension in children with acute lymphoblastic leukemia. Pharmacogenet Genomics.

[69] Kawedia JD, Kaste SC, Pei D, Panetta JC, Cai X, Cheng C, Neale G, Howard SC, Evans WE, Pui CH (2011). Pharmacokinetic, pharmacodynamic, and pharmacogenetic determinants of osteonecrosis in children with acute lymphoblastic leukemia. Blood.

[70] Eden E, Lipson D, Yogev S, Yakhini Z (2007). Discovering motifs in ranked lists of DNA sequences. PLoS Comput Biol.

[71] Eden E, Navon R, Steinfeld I, Lipson D, Yakhini Z (2009). GOrilla: a tool for discovery and visualization of enriched GO terms in ranked gene lists. BMC Bioinformatics.

[72] Bauer-Mehren A, Bundschus M, Rautschka M, Mayer MA, Sanz F, Furlong LI (2011). Gene-disease network analysis reveals functional modules in mendelian, complex and environmental diseases. PLoS One.

[73] Bauer-Mehren A, Rautschka M, Sanz F, Furlong LI (2010). DisGeNET: a Cytoscape plugin to visualize, integrate, search and analyze gene-disease networks. Bioinformatics.

[74] Pinero J, Queralt-Rosinach N, Bravo A, Deu-Pons J, Bauer-Mehren A, Baron M, Sanz F, Furlong LI (2015). DisGeNET: a discovery platform for the dynamical exploration of human diseases and their genes. Database (Oxford).

[75] Queralt-Rosinach N, Pinero J, Bravo A, Sanz F, Furlong LI (2016). DisGeNET-RDF: harnessing the innovative power of the Semantic Web to explore the genetic basis of diseases. Bioinformatics.

[76] 1000 genomes project [http://www.1000genomes.org]

[77] de Bruin RA, McDonald WH, Kalashnikova TI, Yates J, Wittenberg C (2004). Cln3 activates G1-specific transcription via phosphorylation of the SBF bound repressor Whi5. Cell.

[78] Morrison J, Gauderman WJ: Quanto. In*.*, 1.2.4 edn. Los Angeles, CA: USC University of Southern California; 2009.

[79] PLINK. In., 1.07 edn. Cambridge and Boston: Center for Human Genetic Research, Massachusetts General Hospital and the Broad Institute of Harvard and MIT; 2009.

[80] Purcell S, Neale B, Todd-Brown K, Thomas L, Ferreira MA, Bender D, Maller J, Sklar P, de Bakker PI, Daly MJ (2007). PLINK: a tool set for whole-genome association and population-based linkage analyses. Am J Hum Genet.

[81] RStudio. 0.98.1056 ed. Boston: Free Software Foundation INC. p. 2009–13.

[82] IBM SPSS Statistics for Macintosh. In., Version 22.0 edn. Armonk, New-York: IBM; 2013.

[83] Maranon R, Reckelhoff JF (2013). Sex and gender differences in control of blood pressure. Clin Sci (Lond).

[84] Reckelhoff JF (2001). Gender differences in the regulation of blood pressure. Hypertension.

[85] Chow EJ, Pihoker C, Hunt K, Wilkinson K, Friedman DL (2007). Obesity and hypertension among children after treatment for acute lymphoblastic leukemia. Cancer.

[86] Harper RL, Breene RA, Gattens M, Williams RM, Murray MJ (2013). Non-irradiated female survivors of childhood acute lymphoblastic leukaemia are at risk of long-term increases in weight and body mass index. Br J Haematol.

[87] Zhang FF, Rodday AM, Kelly MJ, Must A, MacPherson C, Roberts SB, Saltzman E, Parsons SK (2014). Predictors of being overweight or obese in survivors of pediatric acute lymphoblastic leukemia (ALL). Pediatr Blood Cancer.

[88] Janssen I, Katzmarzyk PT, Ross R (2004). Waist circumference and not body mass index explains obesity-related health risk. Am J Clin Nutr.

[89] Riediger ND, Clara I (2011). Prevalence of metabolic syndrome in the Canadian adult population. CMAJ.

[90] Tomita T (2016). Apoptosis in pancreatic beta-islet cells in Type 2 diabetes. Bosn J Basic Med Sci.

[91] Danial NN, Walensky LD, Zhang CY, Choi CS, Fisher JK, Molina AJ, Datta SR, Pitter KL, Bird GH, Wikstrom JD (2008). Dual role of proapoptotic BAD in insulin secretion and beta cell survival. Nat Med.

[92] Hui H, Dotta F, Di Mario U, Perfetti R (2004). Role of caspases in the regulation of apoptotic pancreatic islet beta-cells death. J Cell Physiol.

[93] Liang Y, Bai G, Doliba N, Buettger C, Wang L, Berner DK, Matschinsky FM (1996). Glucose metabolism and insulin release in mouse beta HC9 cells, as model for wild-type pancreatic beta-cells. Am J Physiol.

[94] Ranger AM, Zha J, Harada H, Datta SR, Danial NN, Gilmore AP, Kutok JL, Le Beau MM, Greenberg ME, Korsmeyer SJ (2003). Bad-deficient mice develop diffuse large B cell lymphoma. Proc Natl Acad Sci U S A.

[95] Datta SR, Ranger AM, Lin MZ, Sturgill JF, Ma YC, Cowan CW, Dikkes P, Korsmeyer SJ, Greenberg ME (2002). Survival factor-mediated BAD phosphorylation raises the mitochondrial threshold for apoptosis. Dev Cell.

[96] Brunham LR, Kruit JK, Hayden MR, Verchere CB (2010). Cholesterol in beta-cell dysfunction: the emerging connection between HDL cholesterol and type 2 diabetes. Current diabetes reports.

[97] Farbstein D, Levy AP (2012). HDL dysfunction in diabetes: causes and possible treatments. Expert Rev Cardiovasc Ther.

[98] Longo LD, Kasper LD, Jameson L, Fauci AS, Hauser LS, Loscalzo J, et al. Diabetes Mellitus. In: Harrison's Principles of Internal Medicine. 18th edition ed. New York: McGraw Hill; 2012.

[99] Gurney JG, Ness KK, Sibley SD, O'Leary M, Dengel DR, Lee JM, Youngren NM, Glasser SP, Baker KS (2006). Metabolic syndrome and growth hormone deficiency in adult survivors of childhood acute lymphoblastic leukemia. Cancer.

[100] Iughetti L, Bruzzi P, Predieri B, Paolucci P (2012). Obesity in patients with acute lymphoblastic leukemia in childhood. Ital J Pediatr.

[101] Siviero-Miachon AA, Spinola-Castro AM, Guerra-Junior G (2009). Adiposity in childhood cancer survivors: insights into obesity physiopathology. Arq Bras Endocrinol Metabol.

[102] Talvensaari KK, Lanning M, Tapanainen P, Knip M (1996). Long-term survivors of childhood cancer have an increased risk of manifesting the metabolic syndrome. J Clin Endocrinol Metab.

[103] Duchatelet S, Caillat-Zucman S, Dubois-Laforgue D, Blanc H, Timsit J, Julier C (2008). FCRL3 -169CT functional polymorphism in type 1 diabetes and autoimmunity traits. Biomed Pharmacother.

[104] Chistiakov DA, Chistiakov AP (2007). Is FCRL3 a new general autoimmunity gene?. Hum Immunol.

[105] Plagnol V, Howson JM, Smyth DJ, Walker N, Hafler JP, Wallace C, Stevens H, Jackson L, Simmonds MJ, Bingley PJ (2011). Genome-wide association analysis of autoantibody positivity in type 1 diabetes cases. PLoS genetics.

[106] Lan W, Fang S, Zhang H, Wang DT, Wu J (2015). The Fc Receptor-Like 3 Polymorphisms (rs7528684, rs945635, rs3761959 and rs2282284) and The Risk of Neuromyelitis Optica in A Chinese Population. Medicine (Baltimore).

[107] Yuan M, Wei L, Zhou R, Bai Q, Wei Y, Zhang W, Huang Y (2016). Four FCRL3 Gene Polymorphisms (FCRL3_3, _5, _6, _8) Confer Susceptibility to Multiple Sclerosis: Results from a Case-Control Study. Mol Neurobiol.

[108] Badawi A, Klip A, Haddad P, Cole DE, Bailo BG, El-Sohemy A, Karmali M (2010). Type 2 diabetes mellitus and inflammation: Prospects for biomarkers of risk and nutritional intervention. Diabetes Metab Syndr Obes.

[109] Wisse BE (2004). The inflammatory syndrome: the role of adipose tissue cytokines in metabolic disorders linked to obesity. J Am Soc Nephrol.

[110] Longo LD, Kasper LD, Jameson L, Fauci AS, Hauser LS, Loscalzo J, et al. Disorders of Lipoprotein Metabolism. In: Harrison's Principles of Internal Medicine. 18th edition edn. New York: McGraw Hill; 2012.

[111] Liu C, Yang J, Han W, Zhang Q, Shang X, Li X, Lu F, Liu X (2015). Polymorphisms in ApoB gene are associated with risk of myocardial infarction and serum ApoB levels in a Chinese population. Int J Clin Exp Med.

[112] Davidson NO (1994). RNA editing of the apolipoprotein B gene A mechanism to regulate the atherogenic potential of intestinal lipoproteins?. Trends Cardiovasc Med.

[113] Skarda J, Amariglio N, Rechavi G (2009). RNA editing in human cancer: review. APMIS.

[114] Makela KM, Seppala I, Hernesniemi JA, Lyytikainen LP, Oksala N, Kleber ME, Scharnagl H, Grammer TB, Baumert J, Thorand B (2013). Genome-wide association study pinpoints a new functional apolipoprotein B variant influencing oxidized low-density lipoprotein levels but not cardiovascular events: AtheroRemo Consortium. Circ Cardiovasc Genet.

[115] Makela KM, Traylor M, Oksala N, Kleber ME, Seppala I, Lyytikainen LP, Hernesniemi JA, Kahonen M, Bevan S, Rothwell PM (2014). Association of the novel single-nucleotide polymorphism which increases oxidized low-density lipoprotein levels with cerebrovascular disease events. Atherosclerosis.

[116] Bolton JL, Hayward C, Direk N, Lewis JG, Hammond GL, Hill LA, Anderson A, Huffman J, Wilson JF, Campbell H (2014). Genome wide association identifies common variants at the SERPINA6/SERPINA1 locus influencing plasma cortisol and corticosteroid binding globulin. PLoS genetics.

[117] Morris AP, Zeggini E (2010). An evaluation of statistical approaches to rare variant analysis in genetic association studies. Genetic epidemiology.

